# Ipsilateral and Contralateral Auditory Brainstem Response Reorganization in Hemispherectomized Patients

**DOI:** 10.1155/2013/832473

**Published:** 2013-12-23

**Authors:** Ning Yao, Hui Qiao, Ping Li, Yang Liu, Liang Wu, Xiaofeng Deng, Zide Wang, Daxing Chen, Xianzeng Tong, Yuan Liu, Chenlong Yang, Yulun Xu

**Affiliations:** ^1^Department of Neurosurgery, Beijing Tiantan Hospital, Capital Medical University, Beijing 100050, China; ^2^Department of Neuroelectrophysiology, Beijing Neurosurgical Institute, Beijing 100050, China; ^3^Department of Neurosurgery, Xuzhou Medical College Affiliated Hospital, Xuzhou Medical College, Jiangsu 221000, China; ^4^Medical Experiments and Testing Center, Capital Medical University, Beijing 100069, China; ^5^Department of Neurosurgery, Beijing Jishuitan Hospital, Beijing 100035, China; ^6^Department of Neurosurgery, The First Teaching Hospital of Xinjiang Medical University, Xinjiang Medical University, Xinjiang 830000, China

## Abstract

*Background*. Cortical hemispherectomy leads to degeneration of ipsilateral subcortical structures, which can be observed long term after the operation. Therefore, reorganization of the brainstem auditory pathway might occur. The aim of this study was to assess reorganization of brainstem auditory pathways by measuring the auditory brainstem response (ABR) in long-term hemispherectomized patients. *Methods*. We performed bilateral monaural stimulation and measured bilateral ABR in 8 patients *~*20 years after hemispherectomy and 10 control subjects. Magnetic resonance imaging (MRI) was performed in patients to assess structural degeneration. *Results*. All patients showed degenerated ipsilateral brainstem structures by MRI but no significant differences in bilateral recording ABR wave latencies. However, nonsurgical-side stimulation elicited significantly longer wave V latencies compared to surgical-side stimulation. Differences in bilateral ABR were observed between hemispherectomized patients and control subjects. Waves III and V latencies elicited by nonsurgical-side stimulation were significantly longer than those in control subjects; surgical-side stimulation showed no significant differences. *Conclusions*. (1) Differences in ABR latency elicited by unilateral stimulation are predominantly due to bilateral brainstem auditory pathway activity rather than to changes in brainstem volume; (2) ABR Waves III and V originate predominantly in the contralateral brainstem; and (3) subcortical auditory pathways appear to reorganize after long term hemispherectomy.

## 1. Introduction

Hemispherectomy is commonly performed for the surgical management of pediatric patients with medically refractory epilepsy [1]. Specific types of hemispherectomy, such as anatomic and functional hemispherectomy, involve removal of diverse areas of the hemisphere. In particular, anatomic hemispherectomy involves complete removal of the cortex from the hemisphere in which the seizures originate [2]. Many studies indicate that reorganization, particularly in the sensorimotor cortex, occurs in the remaining hemisphere [3], leading to recovery of function, depending on how early during postnatal development hemispherectomy is performed. Reorganization of the auditory pathway has also been reported in hemispherectomized patients [4].

Although subcortical structures are left intact in hemispherectomy, degenerative changes can take place and remain long after surgery. In 1966, Oppenheimer and Griffith [5] published an autopsy report of a patient with hemispherectomy, noting degeneration of the midbrain, pons, and other structures ipsilateral to the resected hemisphere. A recent magnetic resonance imaging (MRI) study reported similar findings with respect to degeneration and reorganization of the sensorimotor tracts (pyramidal tract and medial lemniscus) after hemispherectomy [6]. The subcortical auditory pathway, from the acoustic nerve to the lateral geniculate body, ascending through the pons, midbrain, metathalamus, and internal capsule, includes crossed and uncrossed tracts. Similar to the pyramidal tract and medial lemniscus, the auditory pathway might also reorganize in the asymmetric brainstem and remain long after hemispherectomy.

One of the most common tests used to evaluate auditory function in the brainstem is measurement of the auditory brainstem response (ABR). The ABR has been used widely in clinical neurology and is of great value in predicting outcome in patients with severe brain injury, in intraoperative monitoring of cranial nerves, and in evaluating neurologic function [7]. ABR includes five major waves (I–V) that occur within 10 ms after stimulation to one ear. Among these, waves I, III, and V are the most reliable components. Wave I is generated in the peripheral auditory pathway [8], and Waves III and V are believed to arise in the cochlear nuclei and lateral lemniscus, respectively [9]. The latency of ABR waves can provide information regarding change and reorganization in the brainstem auditory pathway.

The aim of the present study was to use MRI to confirm ipsilateral subcortical structural degeneration in patients after long-term hemispherectomy and to assess the bilateral ABR and determine if ABR results can be used to identify reorganization of the auditory pathway.

## 2. Materials and Methods

### 2.1. Subjects

The study protocol was approved by the Beijing Tiantan Hospital Ethics Committee. Subjects provided written informed consent before participating in the present study. Patients were included if they had received hemispherectomy more than 10 years ago and without any relapse of epilepsy regardless of their sex and age, had normal primary neurological and cognitive function (P300) [10], and preserved sensory and motor function [11]. Eight adult patients with long-term hemispherectomy (average, 20.5 years; range, 18–24 years) were tested. The patients included seven men and one woman (27 to 40 years of age at the time of study). Age at seizure onset ranged from birth (congenital) to 6 years. Etiology included intracranial hemorrhage, neonatal jaundice, tuberculous meningitis, hyperpyrexia, and unknown. Preoperative neurologic examinations (computed tomography and electroencephalography) showed various abnormalities as described in a previous study [11]. Age at surgery ranged from 7 to 21 years.

Three patients underwent right-sided operation, and five underwent left-sided operation. All of the patients underwent modified Adams anatomic hemispherectomy. In this procedure, cortical gray and white matter were removed, and the septum pellucidum was left integrated. A muscle flap was placed into the foramen of Monro and sutured to the cerebral falx to chronically separate the subdural space from the cerebral ventricle [11–13]. The basal ganglia and thalamus were completely preserved in all cases. All patients showed contralateral hemiparesis postoperatively but were able to walk unaided and had functional basic social cognitive characteristics to cope with daily life. Seizures disappeared completely after surgery, with no signs of delayed complications, such as hydrocephalus or superficial cerebral hemosiderosis. None of the patients had taken any antiepileptic drugs since hemispherectomy [14]. The pure-tone threshold in these patients was essentially normal (≤25 dB) or very mildly impaired (≤30 dB) [15]. Patient demographic and clinical characteristics are summarized in Table 1.

A total of 10 age-matched, neurologically intact, control subjects without neurologic disease, including seven women and three men, were also assessed. The pure-tone threshold of the control subjects was essentially normal (≤25 dB).

### 2.2. ABR Measurement

ABR measurements were carried out in a low-lit and sound-insulated room with electrical isolation. The temperature was maintained at ~20°C, and relative humidity was maintained within 30% to 75%. A Medelec Synergy Advanced Clinical 10-channel Tower IOM (Oxford Instruments, Abingdon, Oxfordshire, UK) system was utilized. The ABR was elicited with an alternating rarefaction and condensation click stimulus delivered via an unshielded headphone (Synergy N&T Series; Cardinal Health Inc., Madison, WI, USA), with 0.1 ms clicks at a rate of 9.9 click/s. Each trial was performed at an intensity of 80 dB nHL. White-noise masking (40 dB nHL) was performed in the contralateral ear. The ABR was recorded with a bilateral recording system consisting of an active needle electrode attached to the vertex (Cz) and two reference noninverting electrodes attached to the ipsilateral (Mi) and contralateral (Mc) mastoid process, with reference to the recognized 10–20 standard system of the International Federation of EEG Societies. Electrode impedance was <5 kΩ. The filter bandwidth used for recording was 100–3000 Hz. Totals of 1000 to 2000 responses were averaged. The sampling rate was 40 kHz. Each test was carried out 2 or 3 times to ensure that the results were reproducible. Results were recorded both ipsilateral and contralateral to stimulation; for example, after stimulation of the left ear, Cz-Mi and Cz-Mc were recorded concurrently.

The side which received the click stimulation directly is defined as the stimulation side, while the side which the recording electrode is attached to is defined as the recording side. In the control subjects, we used left/right (L/R) side to indicate the two sides; while in the patients, we defined the side of hemispherectomy as the surgical side (S) and the contralateral side as the nonsurgical side (N). Analyzed ABR waveforms included the absolute latency of waves I (L-I), III (L-III), and V (L-V) and the interpeak latency (IPL) of waves I–III (IPL I–III), I–V (IPL I–V), and III–V (IPL III–V). The amplitudes of wave III (A-III) and wave V (A-V) were also analyzed. Relevant abnormalities were defined as prolonged absolute latency or interpeak latency longer than 0.2 ms [16].

### 2.3. Magnetic Resonance Imaging

MRI was performed for all eight patients with a Sigma 3.0 T MRI system (3.0 Tesla; GE Healthcare, Pewaukee, WI, USA). T1- and T2-weighted images were obtained. MR images were routinely taken with serial axial sections of 2 mm thickness.

### 2.4. Statistical Analysis

Statistical analyses were performed with SAS statistical software, version 9.2 (SAS Institute, Inc., Cary, NC, USA). Continuous variables are presented as mean and standard deviation (SD). Repeated measures analysis of variance (ANOVA) with Bonferroni's post hoc tests were used to compare stimulation types and recording side (comparison of 3 or more groups). A paired *t*-test was also used to compare stimulation types and recording side (comparison of two groups). Comparisons between patients and control subjects were analyzed by independent two-sample *t*-test. A two-sided *P* value < 0.05 was considered statistically significant.

## 3. Results

### 3.1. Brainstem Atrophy in Hemispherectomized Patients

**Figure 1:**
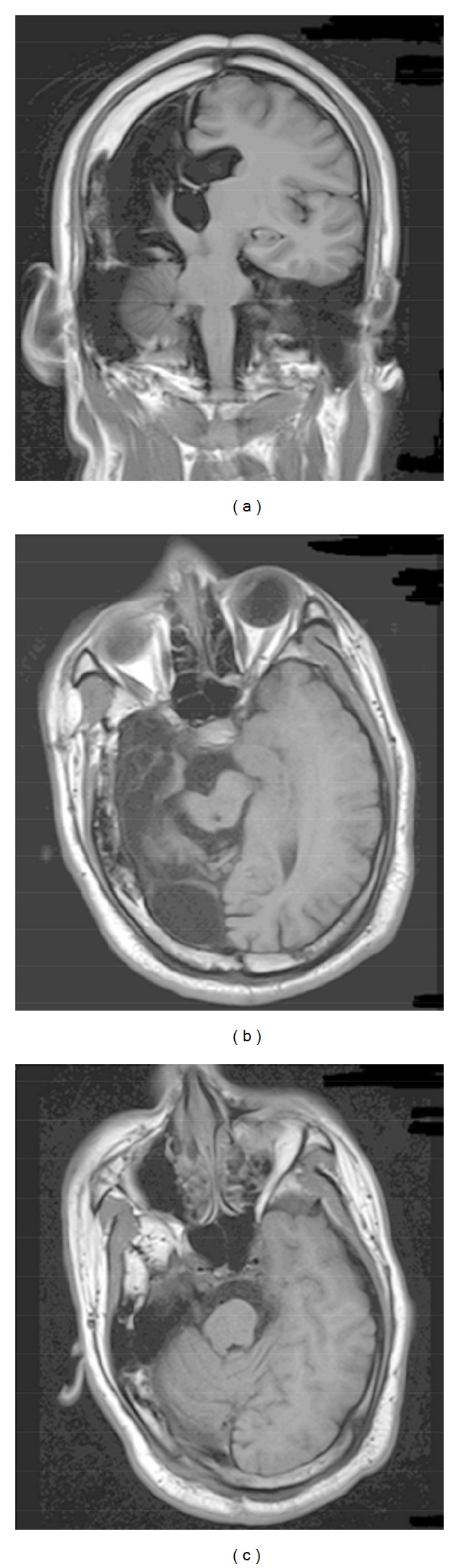
A coronal slice (a) and two axial slices through the midbrain (b) and pons (c) in patient 5. Marked atrophy of the basal nuclei, thalamus, midbrain, and pons ipsilateral to hemispherectomy is shown.

MRI results for all eight hemispherectomized patients showed that the hemisphere on the surgical side was totally resected. In addition, atrophy of the thalamus, basal nuclei, and brainstem (including the midbrain and pons) ipsilateral to hemispherectomy was obvious. The medulla oblongata and cervical cord showed no significant differences compared to the contralateral side (representative results for a single patient are shown in Figure 1).

### 3.2. Absolute Latency, Interpeak Latency, and Wave Amplitude in Control Subjects

Comparisons of absolute latency, interpeak latency, and wave amplitude for the 10 control subjects are summarized in Table 2. There were no statistically significant differences observed in the absolute latencies of Waves I and III. However, the absolute latencies of Wave V recorded on the contralateral sides (L/R, R/L) were longer than that on the ipsilateral side for the R/R group (5.67 ms and 5.66 ms versus 5.55 ms, resp.; *P* ≤ 0.007 for post hoc test). With respect to interpeak latency, IPL III–V waves recorded on the contralateral sides (L/R, R/L) were longer than that recorded on the ipsilateral side for the R/R group (1.96 ms and 1.96 ms versus 1.82 ms, resp.; *P* ≤ 0.008 for post hoc test). With respect to wave III amplitude (A-III), waves recorded on the ipsilateral sides (L/L, R/R) were larger than those on the contralateral sides (L/R, R/L) (0.33 *μ*V and 0.30 *μ*V versus 0.17 *μ*V and 0.17 *μ*V, resp.; *P* ≤ 0.003 for post hoc test). For wave V amplitude (A-V), the amplitude recorded on the ipsilateral side for the R/R group was larger than that on the contralateral side for the L/R group (0.52 *μ*V versus 0.31 *μ*V, resp.; *P* ≤ 0.003).

### 3.3. Absolute Latency, Interpeak Latency, and Wave Amplitude in Hemispherectomized Patients

**Figure 2:**
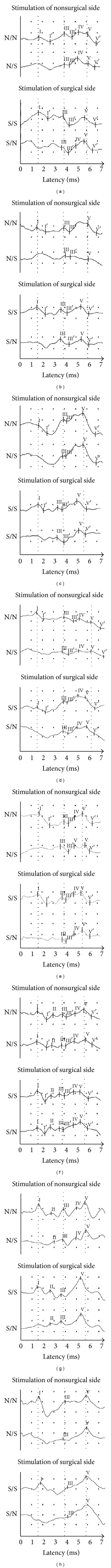
These are the ABR waveforms for all patients. The 3 vertical dotted lines from left to right represent locations of Waves I, III, and V induced by the test stimulation.

Comparisons of absolute latency, interpeak latency, and wave amplitude for the eight hemispherectomized patients are summarized in Table 3 and the ABR waveforms for all eight patients are shown in Figure 2. No significant differences in absolute latency were observed for L-I or L-III. However, the absolute latencies of wave L-V for the N/N and N/S groups were significantly longer than those for the S/S and S/N groups (5.90 ms and 5.97 ms versus 5.56 ms and 5.61 ms, resp.; *P* ≤ 0.005 for post hoc test). With respect to interpeak latency, IPL I–V and IPL I–III for the N/N group were significantly longer than those for the S/S group (IPL I–V: 4.29 ms versus 3.96 ms, resp.; *P* = 0.004; IPL I–III: 2.30 ms versus 2.16 ms, resp.; *P* = 0.041). The IPL III–V wave for the N/S group was significantly longer than that for the S/S group (2.15 ms versus 1.80 ms, resp.; *P* < 0.001). No significant differences were observed for A-III or A-V.

### 3.4. Ipsilateral Side Comparisons between Patients and Control Subjects

Given the lack of significant difference between L/L and R/R sides with respect to absolute latency, interpeak latency, and amplitude in the control subjects, values for the ipsilateral sides (L/L, R/R) were averaged in the control subjects and used to compare with values for the hemispherectomized patients (Table 4). No significant differences in absolute latency between patients and control subjects were observed for L-I. For the N/N patient group, L-III and L-V latencies were significantly longer than those for control subjects (L-III: 3.91 ms versus 3.72 ms, resp.; *P* ≤ 0.002; L-V: 5.90 ms versus 5.56 ms, resp.; *P* ≤ 0.004). No significant differences in L-III or L-V were observed for the S/S patient group compared to control subjects. Similar results were observed for IPL I–V and IPL I–III for the N/N patient group compared to control subjects (IPL I–V: 4.29 ms versus 3.96 ms, resp.; *P* ≤ 0.013; IPL I–III: 2.30 ms versus 2.13 ms, resp.; *P* ≤ 0.001). No significant differences in IPL III–V were observed between patients and control subjects. Significantly lower A-III was observed in the N/N and S/S patient groups compared to control subjects (0.13 *μ*V and 0.15 *μ*V versus 0.32 *μ*V, resp.; *P* ≤ 0.008). No significant differences in A-V were observed between patients and control subjects.

### 3.5. Contralateral Side Comparisons between Patients and Control Subjects

Given the lack of significant difference between L/R and R/L with respect to absolute latency, interpeak latency, and amplitude in the control subjects, values for the contralateral sides (L/R, R/L) in the control subjects were averaged and used to compare with values for the hemispherectomized patients (Table 5). No significant differences in absolute latency between patients and control subjects were observed for L-III. For the N/S patient group, L-V was significantly longer than that for the control group (5.97 ms versus 5.67 ms, resp.; *P* ≤ 0.004). No significant differences were observed in L-III or L-V for the S/N patient group compared to control subjects. Similar results were found for IPL III–V in the N/S patient group (2.15 ms versus 1.96 ms, resp.; *P* ≤ 0.026). No significant differences in A-III or A-V were observed between patients and control subjects.

## 4. Discussion

The aim of the present study was to measure ABR in patients with long-term hemispherectomy and to determine if ABR can be used to identify reorganization of the auditory pathway in such patients. Results showed that whereas all eight of the hemispherectomized patients showed ipsilateral subcortical structural degeneration, as assessed by MRI, they nonetheless showed no significant differences in bilateral recording (N/N versus N/S and S/S versus S/N) ABR wave latencies or amplitudes. However, nonsurgical-side stimulation (N/N, N/S) elicited significantly longer wave V latencies compared to surgical-side stimulation (S/S, S/N). Compared to neurologically intact control subjects, hemispherectomized patients showed significantly longer Wave III and Wave V latencies elicited by nonsurgical-side stimulation (N/N, N/S). No significant differences were seen for Wave III and Wave V latencies elicited by surgical-side stimulation (S/S, S/N).

After long-term hemispherectomy, degeneration of subcortical structures and a large cranial cavity are common phenomena [5, 6]. However, given that bilateral wave latencies (N/N versus N/S and S/S versus S/N) in response to monaural stimulation were not significantly different in the presence of these structural changes in our patients indicates that based on the volume conductor effect, brain volume was not a major factor in the conduction of auditory evoked potentials [17]. Therefore, significant differences in ABR in the hemispherectomized patients are thought to be due to reorganization of the brainstem auditory pathways (crossed and uncrossed pathways). Distortion of auditory pathways and/or demyelination of contralateral pathways may be part of the explanation for our findings.

ABR is generally recorded from the side ipsilateral to stimulation. Contralateral recording is not thought to contribute significantly to lesion detection; the main value is to aid in the recognition of certain waves when they are not clearly visible in the ipsilateral recording [18]. Hatanaka and associates [19] suggested that contralateral recordings can be a useful measure of developmental changes in the infant auditory pathway. Our present results showed no significant differences in ABR wave latencies elicited by ipsilateral stimulation (L/L, R/R) in control subjects. However, Wave V latencies showed slight but statistically significant increases in response to contralateral stimulation (L/R, R/L). These findings are similar to those of other reports [20–23]. In addition, Wave III showed significantly smaller amplitudes in response to contralateral (L/R, R/L) versus ipsilateral (L/L, R/R) stimulation in control subjects. The reason for this difference is not known. Possible explanations include methodologic recording differences [20] or lateralization of the brainstem auditory pathway [23].

Detailed information regarding sites of generation of ABR responses would further enhance the clinical applicability and relevance of ABR. Among the three major waves (Waves I, III, and V), Wave I has been demonstrated to originate from the ipsilateral auditory nerve or at its point of entry to the brainstem [24]. Møller and Jannetta [25] suggested that Wave III is associated with activity in or near the ipsilateral cochlear nucleus. Wave III is also associated with activity of the superior olivary complex [9, 26]. Wave V has been attributed to activity in the contralateral lateral lemniscus, which terminates in the inferior colliculus [9, 24, 27]. ABR designation of sites of generation remains to be fully elucidated. Contrary to the point of view of Møller and associates [9] that Wave III originates ipsilaterally and Wave V originates contralaterally, studies of patients with brainstem lesions suggest that the ipsilateral auditory pathway might be the main generator of wave V [28]. Casali and Dos Santos [29] reviewed the literature and stated that both Waves III and V receive contralateral inputs probably in greater number than ipsilateral inputs. The same conclusion was made by Strauss and associates [30]. With respect to the hemispherectomized patients in our study, we suggest that the resected hemisphere resulted in retrodegeneration of the ipsilateral subcortical nucleus in the brainstem auditory pathway. Degeneration of an auditory nucleus might result in prolongation of ABR latency in response to nonsurgical-side stimulation (N/N, N/S) compared to control subjects.

Acoustic information from each ear ascends ipsilaterally and contralaterally to reach both auditory cortices [4]. However, it has been reported that the contralateral pathway has a marked advantage over the ipsilateral pathway [4, 31, 32]. The longer latencies of Waves III and V elicited by stimulation of the nonsurgical side in patients indicate that the contralateral brainstem contributes to Waves III and V [29]. Prolongation of wave V would be related to the marked degeneration of the superior pons and that of Wave III would be related to degeneration of the inferior pons.

Although the auditory function of patients in our study was not severely affected by hemispherectomy, there is a report of impaired sound localization in hemispherectomized patients [32]. Those authors suggest that a single hemisphere and/or residual (subcortical) structures cannot analyze sound localization as efficiently as two fully functional hemispheres. In addition, de Bode and associates [31] tested dichotic listening in hemispherectomized patients and suggested that language function is affected by the absence of a hemisphere, citing specialization of the cortical hemispheres for language. Both reports suggested that after hemispherectomy, function of the crossed auditory pathway performs better than that of the uncrossed pathway; the ear ipsilateral to hemispherectomy shows better function than the ear contralateral to hemispherectomy. These studies are similar to our results in that the ABR latencies elicited from the surgical side (S/S, S/N) were shorter than those elicited from the nonsurgical side (N/N, N/S). Both of the above reports [31, 32] focused on the hemispheric cortex, ignoring subcortical auditory structures such as the inferior colliculus and lateral lemniscus, which also provide a coding mechanism for locating sources in space [33]. The superior olivary complex of the mammalian brainstem is also involved in computing sound localization [34]. Our present observation of significant differences in bilateral ABR wave latencies in hemispherectomized patients indicates that the impaired auditory function occurs, at least in part, at the subcortical level.

It is important to understand the implications of significant differences in bilateral ABR wave latency in hemispherectomized patients. An auditory functional MRI study on hemispherectomized subjects demonstrated significantly decreased activity in the intact hemisphere in response to monaural stimulation of surgical side and increased activity in response to stimulation of normal side [4]. The authors suggested that a substantial amount of functional reorganization takes place subcortically. Studies have also indicated that after hemispherectomy or diffuse cortical injury, crossed and uncrossed auditory pathways undergo functional reorganization [31, 32, 35]. In the present study, even though significant differences in bilateral ABR were observed in hemispherectomized patients, all of the waveforms could be elicited, similar to the control subjects. Thus, we believe that auditory function of the brainstem remains normal rather than being impaired by hemispherectomy. In addition, differences in bilateral ABR latency in hemispherectomized patients indicate that reorganization may take place in the brainstem auditory pathways. Our results also indicate that the prolonged latency of Wave V was greater than that of Wave III and thus suggest that reorganization of the subcortical auditory pathways may take place mainly in the lateral lemniscus, between the level of the superior olivary complex and the inferior colliculus. We suggest that in the event of severe injury to the cortex, auditory function of the subcortical crossed pathway might be impaired and therefore reinforced by the uncrossed pathway. The intact hemisphere, along with its ascending pathway, might also undergo changes. Further studies combining high-resolution neuroimaging and animal histology are needed to elucidate the exact nature of auditory pathway reorganization after hemispherectomy.

Potential limitations of the present study include the fact that whereas all but one of the hemispherectomized patients were male, most of the control subjects (7/10) were female. There is a report suggesting that ABR latency and amplitude can differ according to sex [36]; however, other research indicates that this difference might be small enough to be nonsignificant [37].

In conclusion, the use of bilateral ABR measurements in response to monaural stimulation was able to identify prolonged latencies in Wave III, thought to arise in the cochlear nuclei, and Wave V, thought to arise in the lateral lemniscus, in response to nonsurgical-side (i.e., contralateral to hemispherectomy) stimulation versus surgical-side (ipsilateral to hemispherectomy) stimulation in hemispherectomized patients compared to control subjects. The use of ABR in this clinical situation may be helpful in further elucidating subcortical reorganization of the auditory pathways. Our present results indicate that (1) differences in bilateral ABR latency elicited from monaural stimulation are due mainly to bilateral brainstem auditory pathway activity rather than to changes in brain volume; (2) ABR Waves III and V originate mainly in the contralateral brainstem; and (3) subcortical auditory structures are involved in the functional reorganization of the auditory pathways after hemispherectomy.

## Figures and Tables

**Table 1:** Demographic and clinical characteristics of hemispherectomized patients.

Subject	Sex	Age (year)	Removed hemisphere	Aetiology	Age of first seizure (year.month)	Age of definite diagnosis of epilepsy (year.month)	Age at operation (year)	Pure-tone thresholds (dB)	
								Left	Right
1	Male	34	Left	Intracranial hemorrhage	6.0	6.0	10	20	30
2	Male	36	Left	Intracranial hemorrhage	0.1	0.1	15	15	15
3	Male	31	Left	Neonatal jaundice	After birth	4.0	8	15	20
4	Female	40	Left	Tuberculous meningitis	0.9	9.0	21	15	15
5	Male	27	Right	Unknown	After birth	0.2	9	10	15
6	Male	29	Right	Intracranial hemorrhage	0.2	0.2	11	5	15
7	Male	33	Left	Hyperpyrexia	0.1	4.0	12	20	20
8	Male	27	Right	Unknown	5.0	5.0	7	30	20

**Table 2:** Absolute latency, interpeak latency, and wave amplitude in control subjects.

	Stimulation side/recording side				*P* value
	L/L (*n* = 10)	R/R (*n* = 10)	L/R (*n* = 10)	R/L (*n* = 10)	
	Mean ± SD	Mean ± SD	Mean ± SD	Mean ± SD	
Latency (ms)					
L-I	1.58 ± 0.14	1.61 ± 0.19	—	—	0.407
L-III	3.72 ± 0.13	3.73 ± 0.15	3.71 ± 0.17	3.70 ± 0.15	0.868
L-V	5.57 ± 0.20	5.55 ± 0.21	5.67 ± 0.17^‡^	5.66 ± 0.21^‡^	0.010*
Interpeak latency (ms)					
IPL I–V	3.99 ± 0.23	3.94 ± 0.17	—	—	0.384
IPL I–III	2.14 ± 0.12	2.11 ± 0.12	—	—	0.595
IPL III–V	1.85 ± 0.15	1.82 ± 0.13	1.96 ± 0.15^‡^	1.96 ± 0.13^‡^	0.004*
Amplitude (*μ*V)					
A-III	0.33 ± 0.09	0.30 ± 0.10	0.17 ± 0.11^†‡^	0.17 ± 0.10^†‡^	<0.001*
A-V	0.37 ± 0.13	0.52 ± 0.19	0.31 ± 0.10^‡^	0.39 ± 0.18	0.003*

A: amplitude; IPL: interpeak latency; L: absolute latency; L: left side; R: right side; —: wave not detectable.

*P < 0.05 between the four groups.

^†^Significant difference compared to L/L.

^‡^Significant difference compared to R/R.

**Table 3:** Absolute latency, interpeak latency, and wave amplitude in hemispherectomized patients.

	Stimulation side/recording side				*P* value
	N/N (*n* = 8)	S/S (*n* = 8)	N/S (*n* = 8)	S/N (*n* = 8)	
	Mean ± SD	Mean ± SD	Mean ± SD	Mean ± SD	
Latency (ms)					
L-I	1.61 ± 0.09	1.60 ± 0.10	—	—	0.660
L-III	3.91 ± 0.07	3.75 ± 0.24	3.82 ± 0.14	3.72 ± 0.25	0.087
L-V	5.90 ± 0.24	5.56 ± 0.27^†^	5.97 ± 0.21^‡^	5.61 ± 0.22^†§^	<0.001*
Interpeak latency (ms)					
IPL I–V	4.29 ± 0.31	3.96 ± 0.24	—	—	0.004*
IPL I–III	2.30 ± 0.09	2.16 ± 0.20	—	—	0.041*
IPL III–V	1.99 ± 0.23	1.80 ± 0.11	2.15 ± 0.21^‡^	1.89 ± 0.14	<0.001*
Amplitude (*μ*V)					
A-III	0.13 ± 0.07	0.15 ± 0.14	0.11 ± 0.13	0.21 ± 0.18	0.265
A-V	0.61 ± 0.38	0.61 ± 0.42	0.46 ± 0.38	0.42 ± 0.23	0.190

A: amplitude; IPL: interpeak latency; L: absolute latency; N: nonsurgical side; S: surgical side; —: wave not detectable.

*P < 0.05 between the four groups.

^†^Significant difference compared to N/N.

^‡^Significant difference compared to S/S.

^§^Significant difference compared to N/S.

**Table 4:** Ipsilateral side comparisons between patients and control subjects.

	Control subjects (*n* = 10)	Patients (*n* = 8)		*P* value compared with N/N versus control	*P* value compared with S/S versus control
	Averaged L/L and R/R	N/N	S/S		
	Mean ± SD	Mean ± SD	Mean ± SD		
Latency (ms)					
L-I	1.60 ± 0.15	1.61 ± 0.09	1.60 ± 0.10	0.816	0.994
L-III	3.72 ± 0.13	3.91 ± 0.07	3.75 ± 0.24	0.002*	0.739
L-V	5.56 ± 0.19	5.90 ± 0.24	5.56 ± 0.27	0.004*	0.971
Interpeak latency (ms)					
IPL I–V	3.96 ± 0.19	4.29 ± 0.31	3.96 ± 0.24	0.013*	0.973
IPL I–III	2.13 ± 0.10	2.30 ± 0.09	2.16 ± 0.20	0.001*	0.667
IPL III–V	1.84 ± 0.12	1.99 ± 0.23	1.80 ± 0.11	0.089	0.555
Amplitude (*μ*V)					
A-III	0.32 ± 0.08	0.13 ± 0.07	0.15 ± 0.14*	<0.001*	0.008
A-V	0.45 ± 0.13	0.61 ± 0.38	0.61 ± 0.42	0.292	0.330

A: amplitude; IPL: interpeak latency; L: absolute latency; L: left side; N: nonsurgical side; R: right side; S: surgical side.

*P < 0.05 compared to control subjects.

**Table 5:** Contralateral-side comparisons between patients and control subjects.

	Control subjects (*n* = 10)	Patients (*n* = 8)		*P* value compared with N/S versus control	*P* value compared with S/N versus control
	Averaged L/R and R/L	N/S	S/N		
	Mean ± SD	Mean ± SD	Mean ± SD		
Latency (ms)					
L-III	3.70 ± 0.15	3.82 ± 0.14	3.72 ± 0.25	0.106	0.870
L-V	5.67 ± 0.18	5.97 ± 0.21	5.61 ± 0.22	0.004*	0.550
Interpeak latency (ms)					
IPL III–V	1.96 ± 0.12	2.15 ± 0.21	1.89 ± 0.14	0.026*	0.249
Amplitude (*μ*V)					
A-III	0.17 ± 0.09	0.11 ± 0.13	0.21 ± 0.18	0.291	0.520
A-V	0.35 ± 0.12	0.46 ± 0.38	0.42 ± 0.23	0.468	0.460

A: amplitude; IPL: interpeak latency; L: absolute latency; L: left side; N: nonsurgical side; R: right side; S: surgical side.

*P < 0.05 compared to control subjects.

## References

[1] Fountas KN, Smith JR, Robinson JS, Tamburrini G, Pietrini D, Di Rocco C (2006). Anatomical hemispherectomy. *Child’s Nervous System*.

[2] de Almeida AN, Marino R, Aguiar PH, Teixeira MJ (2006). Hemispherectomy: a schematic review of the current techniques. *Neurosurgical Review*.

[3] Holloway V, Gadian DG, Vargha-Khadem F, Porter DA, Boyd SG, Connelly A (2000). The reorganization of sensorimotor function in children after hemispherectomy. A functional MRI and somatosensory evoked potential study. *Brain*.

[4] Paiement P, Champoux F, Bacon BA (2008). Functional reorganization of the human auditory pathways following hemispherectomy: an fMRI demonstration. *Neuropsychologia*.

[5] Oppenheimer DR, Griffith HB (1966). Persistent intracranial bleeding as a complication of hemispherectomy. *Journal of Neurology Neurosurgery and Psychiatry*.

[6] Choi JT, Vining EPG, Mori S, Bastian AJ (2010). Sensorimotor function and sensorimotor tracts after hemispherectomy. *Neuropsychologia*.

[7] Soldner F, Hölper BM, Choné L, Wallenfang T (2001). Evoked potentials in acute head injured patients with MRI-detected intracerebral lesions. *Acta Neurochirurgica*.

[8] Oh SJ, Kuba T, Soyer A, Choi IS, Bonikowski FP, Vitek J (1981). Lateralization of brainstem lesions by brainstem auditory evoked potentials. *Neurology*.

[9] Moller AR, Jho HD, Yokota M, Jannetta PJ (1995). Contribution from crossed and uncrossed brainstem structures to the brainstem auditory evoked potentials: a study in humans. *Laryngoscope*.

[10] Tong XZ, Xu YL, Fu Z (2009). Long-term P300 in hemispherectomized patients. *Chinese Medical Journal*.

[11] Chen BH (1989 (Chinese)). *Functional and Stereotactic Neurosurgery*.

[12] Adams CBT (1983). Hemispherectomy: a modification. *Journal of Neurology Neurosurgery and Psychiatry*.

[13] Xu Y, Li S, Chen B (1997). Long-term results in 31 patients on total hemispherectomy modified for infantile hemiplegic epilepsy. *Zhonghua Wai Ke Za Zhi*.

[14] Yao N, Qiao H, Shu N (2013). Cortex mapping of ipsilateral somatosensory area following anatomical hemispherectomy: a MEG study. *Brain and Development*.

[15] Kutz JW Audiology pure-tone testing. http://emedicine.medscape.com/article/1822962-overview#a01.

[16] Shih C, Tseng FY, Yeh TH, Hsu CJ, Chen YS (2009). Ipsilateral and contralateral acoustic brainstem response abnormalities in patients with vestibular schwannoma. *Otolaryngology—Head and Neck Surgery*.

[17] Pan YF (2000 (Chinese)). *Evoked Potentials in Medical Practice*.

[18] Hammond SR, Yiannikas C (1987). The relevance of contralateral recordings and patient disability to assessment of brain-stem auditory evoked potential abnormalities in multiple sclerosis. *Archives of Neurology*.

[19] Hatanaka T, Shuto H, Yasuhara A, Kobayashi Y (1988). Ipsilateral and contralateral recordings of auditory brainstem responses to monaural stimulation. *Pediatric Neurology*.

[20] Kato T, Shiraishi K, Imamura A, Kimura K, Morizono T, Soda T (1995). Analysis of auditory brainstem response waveforms derived ipsilaterally and contralaterally to monaural stimulation. *Auris Nasus Larynx*.

[21] Prasher DK (1995). Differential effect of adaptation on the ipsi- and contralateral auditory brainstem pathways. *Scandinavian Audiology*.

[22] Rosenhamer H, Holmkvist C (1982). Bilaterally recorded auditory brainstem responses to monaural stimulation. Latency and amplitude differences in normal subjects. *Scandinavian Audiology*.

[23] Han D, Xu S (2004 (Chinese)). *Basic and Clinical Audiology*.

[24] Parkkonen L, Fujiki N, Mäkelä JP (2009). Sources of auditory brainstem responses revisited: contribution by magnetoencephalography. *Human Brain Mapping*.

[25] Møller AR, Jannetta PJ (1983). Auditory evoked potentials recorded from the cochlear nucleus and its vicinity in man. *Journal of Neurosurgery*.

[26] Stone JL, Calderon-Arnulphi M, Watson KS (2009). Brainstem auditory evoked potentials—a review and modified studies in healthy subjects. *Journal of Clinical Neurophysiology*.

[27] Voordecker P, Brunko E, de Beyl Z (1988). Selective unilateral absence or attenuation of wave V of brain-stem auditory evoked potentials with intrinsic brain-stem lesions. *Archives of Neurology*.

[28] Markand ON, Farlow MR, Stevens JC, Edwards MK (1989). Brain-stem auditory evoked potential abnormalities with unilateral brain-stem lesions demonstrated by magnetic resonance imaging. *Archives of Neurology*.

[29] Casali RL, Dos Santos MFC (2010). Auditory Brainstem evoked response: response patterns of full-term and premature infants. *Brazilian Journal of Otorhinolaryngology*.

[30] Strauss C, Naraghi R, Bischoff B, Huk WJ, Romstöck J (2000). Contralateral hearing loss as an effect of venous congestion as the ipsilateral inferior colliculus after microvascular decompression: report of a case. *Journal of Neurology Neurosurgery and Psychiatry*.

[31] de Bode S, Sininger Y, Healy EW, Mathern GW, Zaidel E (2007). Dichotic listening after cerebral hemispherectomy: methodological and theoretical observations. *Neuropsychologia*.

[32] Lessard N, Lepore F, Poirier P, Villemagne J, Lassonde M (2000). Sound localization in hemispherectomized subjects: the contribution of crossed and uncrossed cortical afferents. *Experimental Brain Research*.

[33] Litovsky RY, Fligor BJ, Tramo MJ (2002). Functional role of the human inferior colliculus in binaural hearing. *Hearing Research*.

[34] Tzounopoulos T, Kraus N (2009). Learning to encode timing: mechanisms of plasticity in the auditory brainstem. *Neuron*.

[35] Clarkson C, López DE, Merchán MA (2010). Long-term functional recovery in the rat auditory system after unilateral auditory cortex ablation. *Acta Oto-Laryngologica*.

[36] McFadden D, Hsieh MD, Garcia-Sierra A, Champlin CA (2010). Differences by sex, ear, and sexual orientation in the time intervals between successive peaks in auditory evoked potentials. *Hearing Research*.

[37] Chiappa KH (1990). *Evoked Potentials in Clinical Medicine*.

